# Trends and Disparities in Cancer‐Related Mortality Among Adults With Diabetes in the United States: 1999–2019

**DOI:** 10.1002/edm2.70092

**Published:** 2025-09-25

**Authors:** Muhammad Saad, Dua Ali, Taimor Mohammed Khan, Ruqiat Masooma Batool, Muhammad Sameer Arshad, Peter Collins, Raheel Ahmed

**Affiliations:** ^1^ Department of Medicine Dow University of Health Sciences Karachi Pakistan; ^2^ Baylor Scott and White Research Institute Dallas Texas USA; ^3^ National Heart and Lung Institute Imperial College London London UK

## Abstract

**Aim:**

Cancer and diabetes are major public health concerns, with diabetes linked to increased cancer‐related mortality. However, national trends and disparities remain underexplored.

**Methods:**

Using CDC WONDER data, we analysed deaths where both diabetes and cancer were listed as causes. Age‐adjusted mortality rates (AAMRs) were calculated for diabetic cancer patients aged ≥ 25 years and stratified by demographics and geography. Joinpoint regression estimated annual percent changes (APCs) and average annual percent changes (AAPCs).

**Results:**

From 1999 to 2019, 699,007 cancer‐related deaths occurred among individuals with diabetes. The overall AAMR increased from 15.06 to 15.23 per 100,000 (AAPC: +0.07%; *p* = 0.20), with a rise from 1999 to 2003, a decline from 2003 to 2015, and a resurgence from 2015 to 2019. Men (AAMR: 20.83) had higher mortality than women (AAMR: 11.80). Non‐Hispanic Black individuals had the highest AAMRs (23.72), but NH American Indian/Alaska Natives had the largest increase (AAPC: 0.60). The Midwest (AAMR: 17.03) and rural areas (AAMR: 18.70) had the highest mortality, with rural rates rising significantly (AAPC: 0.92). Gastrointestinal cancers were the leading cause (AAMR: 4.31), followed by haematological (AAMR: 1.80), prostate (AAMR: 1.59), and breast cancer (AAMR: 1.38).

**Conclusion:**

Cancer‐related mortality in individuals with diabetes has increased, with notable disparities. Targeted interventions, screening, and better diabetes management are essential to reducing risks in high‐risk populations.

## Graphical abstract

From 1999 to 2019, cancer‐related mortality in diabetics showed fluctuating trends with rising rates in rural areas and specific ethnic groups. Men, Black individuals, and those in the Midwest had higher mortality. Disparities highlight the urgent need for targeted prevention, screening, and diabetes management interventions.

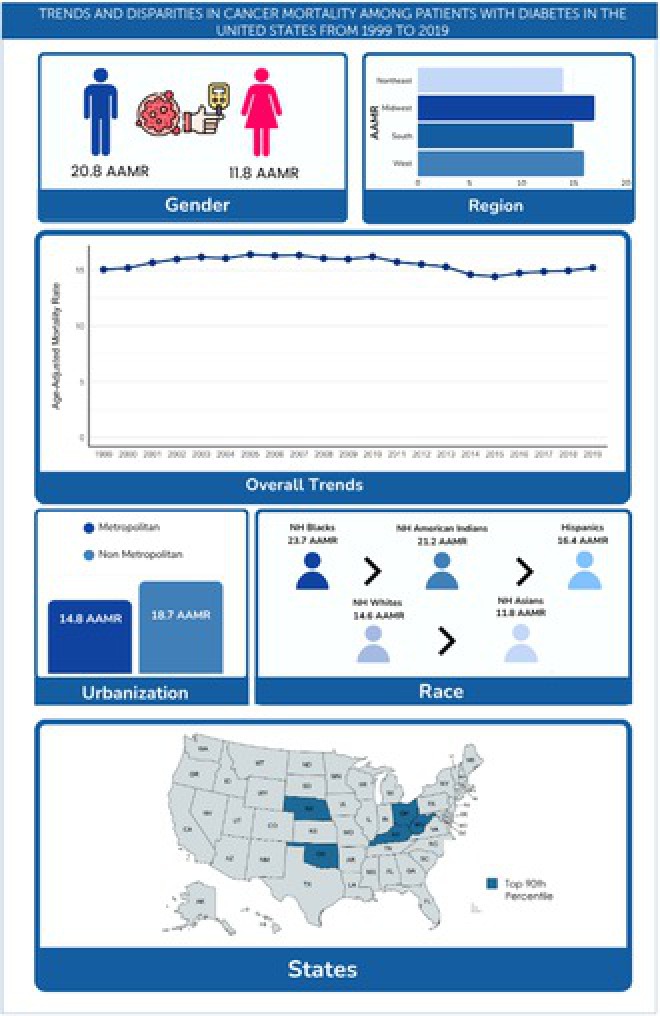

## 1 Introduction

Cancer remains a leading cause of morbidity and mortality worldwide, with an estimated 19.3 million new cases and nearly 10 million cancer‐related deaths reported in 2020 alone [1]. In the United States, cancer is the second leading cause of death, accounting for approximately 600,000 fatalities annually [2]. Concurrently, the prevalence of diabetes mellitus has surged, affecting over 34 million Americans, or roughly 1 in 10 adults [3].

The cost burden of managing cancer and diabetes in the United States is substantial. Diabetes alone incurs an annual cost of approximately $412.9 billion [4], while cancer care costs reached about $208.9 billion in 2020 [5], highlighting the significant financial impact of these chronic conditions on patients and the healthcare system.

Research indicates that adults with diabetes have a 20%–30% higher risk of cancer‐related mortality compared to their non‐diabetic counterparts [6]. This association is attributed to shared risk factors such as obesity, inflammation, and metabolic dysregulation, which can exacerbate cancer progression and complicate treatment outcomes [7, 8]. Furthermore, the burden of cancer is disproportionately felt among racial and ethnic minorities, who often experience higher rates of both diabetes and cancer, leading to significant health disparities [9]. The coexistence of cancer and diabetes presents a significant public health challenge, as individuals with diabetes are at an increased risk of developing various malignancies, including pancreatic, liver, and colorectal cancers [10].

Understanding the trends and disparities in cancer‐associated mortality among adults with diabetes is crucial in improving public health strategies and health equity. This study aims to analyse cancer‐associated mortality trends in adults with diabetes in the United States from 1999 to 2019, utilising data from the CDC WONDER database. By examining variations based on sex, race/ethnicity, geographic region, and urban–rural distinctions, we aim to support the development of targeted interventions to address the unique challenges faced by this vulnerable population.

## 2 Methodology

### 2.1 Study Setting and Population

This study utilised data from the CDC WONDER database, spanning January 1, 1999, to December 31, 2019 [11]. Death certificate records from the Multiple Cause‐of‐Death Public Use files were examined to identify cases where both diabetes and cancer were listed as underlying or contributing causes of death. Diabetes‐related deaths were identified using ICD‐10 codes E10‐E14, while cancer‐related deaths were classified under ICD‐10 codes C00–C97 [12, 13, 14, 15]. The study population included individuals aged 25 years and older. Since the dataset consisted of publicly available, de‐identified information provided by a governmental source, institutional review board (IRB) approval was not required.

### 2.2 Data Abstraction

We retrieved mortality data for individuals with both diabetes and cancer, capturing demographic variables, population estimates, year of death, and geographic distribution. Additional variables included urban–rural status, state‐level data, regional divisions, and place of death (hospital, home, hospice, or long‐term care facility). Urban and rural classifications were based on the National Center for Health Statistics Urban–Rural Classification Scheme, while regional categories followed U.S. Census Bureau divisions: Northeast, Midwest, South, and West [16]. Racial and ethnic classifications were recorded according to U.S. Office of Management and Budget standards, encompassing non‐Hispanic (NH) White, NH Black, Hispanic or Latino, NH American Indian or Alaska Native, and NH Asian [11]. Mortality trends were further analysed across three age groups: young adults (25–44 years), middle‐aged adults (45–64 years), and older adults (65 years and above) [17]. To examine mortality patterns by cancer subtype, we focused on five commonly occurring cancer types: lung (C34), gastrointestinal (GI) (C15–C26), prostate (C61), breast (C50), and hematologic malignancies (C81–C96), reflecting the most prevalent cancers based on national statistics [18].

### 2.3 Statistical Analysis

To evaluate national mortality trends related to diabetes and cancer, both crude mortality rates and age‐adjusted mortality rates (AAMRs) per 100,000 population were calculated from 1999 to 2019. These rates were stratified by year, sex, race, state, region, and age group. Crude mortality rates were determined by dividing the number of diabetes and cancer‐related deaths by the corresponding U.S. population for each year, while AAMRs were computed by standardising mortality rates to the 2000 U.S. population [19]. Confidence intervals (95% CIs) were reported for all estimates. Temporal trends were assessed using the Joinpoint Regression Program (Version 5.2.0, National Cancer Institute), which applies log‐linear regression models to detect significant shifts in trends over time [20]. Sensitivity analyses were conducted by considering cases where cancer was the underlying cause of death (UCD) and diabetes was a multiple cause of death (MCD). To determine annual trends in diabetes and cancer‐related mortality, we calculated annual percent change (APC) and average annual percent change (AAPC), along with their respective 95% CIs. A trend was classified as increasing or decreasing if the slope significantly deviated from zero, with statistical significance evaluated using two‐tailed *t*‐tests (*p* ≤ 0.05). Parallelism tests were performed to assess whether trends in AAMRs for cancer‐related mortality differed significantly from those for combined cancer and diabetes‐related mortality. Additionally, we examined whether trends in AAMRs for diabetes‐related mortality were statistically distinct from those for combined cancer and diabetes‐related mortality. A significant *p*‐value in the interaction test indicated that the respective trends, when analysed using AAPC, were statistically different.

## 3 Results

Between 1999 and 2019, a total of 699,007 cancer‐associated deaths were recorded among adults (≥ 25 years old) with diabetes. Of these, 33.29% occurred in medical facilities, 20.68% in nursing homes/long‐term care facilities, 5.10% in hospices, 36.38% at home, and 4.54% in other locations (Table 1).

**TABLE 1:** Frequency and age‐adjusted mortality rates per 100,000 in adults with concomitant cancer and diabetes stratified by age group, sex, race, census region, urbanisation and location of death.

	Deaths	Population	AAMR 1999 (95% CI)	AAMR 2019 (95% CI)	AAPC (95% CI)
Overall	699,007	4,247,219,476	15.06 (14.88–15.24)	15.23 (15.08–15.38)	0.07 (−0.05 to 0.22)
Age group^a^					
Young adults (25–44 years)	4452	1,763,170,919	0.23 (0.20–0.26)	0.25 (0.25–0.26)	0.71 (−0.05 to 1.53)
Middle‐aged adults (45–64 years)	123,031	1,611,231,257	7.02 (6.81–7.23)	8.35 (8.15–8.55)	0.95* (0.79 to 1.13)
Older adults (65+ years)	571,524	8,728,173,000	63.70 (62.86–64.54)	62.03 (61.36–62.69)	−0.13* (−0.20 to −0.04)
Sex					
Men	394,678	2,044,831,030	20.00 (19.67–20.34)	20.66 (20.39–20.92)	0.19* (0.05 to 0.41)
Women	304,329	2,202,388,446	11.84 (11.63–12.05)	11.07 (10.90–11.24)	−0.28* (−0.53 to −0.20)
NH race					
NH American Indian/Alaska Native	5073	31,309,861	18.13 (14.91–21.35)	23.59 (21.19–25.99)	0.60 (−0.04–1.35)
NH White	524,591	2,949,005,634	13.93 (13.74–14.12)	14.72 (14.55–14.89)	0.28* (0.18–0.40)
NH Black/African American	95,384	490,967,596	25.97 (25.14–26.80)	20.44 (19.87–21.01)	−1.17* (−1.37 to −0.91)
NH Asian/Pacific Islander	19,372	222,344,368	12.23 (11.10–13.36)	10.54 (9.98–11.10)	−0.44 (−0.84 to 0.31)
Hispanic race	53,181	553,592,017	15.72 (14.85–16.59)	15.18 (14.68–15.69)	−0.13 (−0.39 to 0.19)
Census region					
Northeast	124,871	787,775,220	15.60 (15.20–16.01)	12.10 (11.79–12.42)	−1.34* (−1.63 to −1.10)
Midwest	172,929	922,845,632	16.72 (16.33–17.11)	15.59 (15.26–15.91)	−0.40* (−0.59 to −0.23)
South	247,404	1,565,644,413	13.76 (13.47–14.05)	16.08 (15.83–16.33)	0.83* (0.67–1.05)
West	153,803	970,954,211	14.61 (14.21–15.01)	15.88 (15.55–16.20)	0.31* (0.07–0.66)
Urbanisation					
Metropolitan	548,943	3,600,613,712	14.66 (14.46–14.86)	14.27 (14.12–14.43)	−0.16 (−0.28 to 0.02)
Nonmetropolitan	150,064	646,605,764	16.60 (16.17–17.04)	19.85 (19.43–20.26)	0.92* (0.77–1.13)
Location of death					
Medical facility	232,677	—	—	—	—
Hospice facility	35,650	—	—	—	—
Nursing home/long term care	144,579	—	—	—	—
Decedent's home	254,319	—	—	—	—

Abbreviations: AAMR, age‐adjusted mortality rate; AAPC, average annual percent change; NH, Non‐Hispanic.

^a^ Crude mortality rates were used instead of AAMRs for Age Groups.

* Indicates level of statistical significance *p* ≤ 0.05.

### 3.1 Annual Trends for Cancer‐Associated AAMR in Patients With Diabetes

**FIGURE 1:**
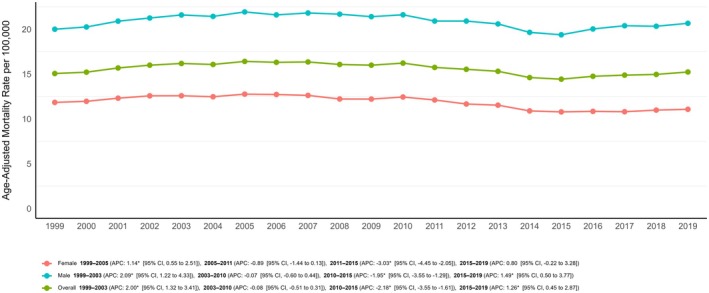
Overall and sex‐based trends in diabetes and cancer‐related mortality rates in adults in the United States from 1999 to 2019.

The AAMR for cancer‐related deaths in adults over 25 with diabetes was 15.06 (95% CI: 14.88–15.24) in 1999 and rose to 15.23 (95% CI: 15.48–15.55) by 2019 (AAPC: +0.07%; 95% CI: −0.05 to 0.22; *p* = 0.20). The overall AAMR showed a steady increase between 1999 and 2003 (APC: +2.00%; 95% CI: 1.32 to 3.41; *p* < 0.001) followed by a plateau phase till 2010 (APC: −0.08%; 95% CI: −0.51 to 0.31; *p* = 0.61), then a gradual decrease until 2015 (APC: −2.18%; 95% CI: −3.55 to −1.61; *p* < 0.001) and a steady rise until 2019 (APC: +1.26%; 95% CI: 0.45 to 2.87; *p* = 0.004) (Table 1; Figure 1; Table S2).

Sensitivity analysis revealed a similar trend in AAMRs when cancer was considered as the underlying cause of death and diabetes as a contributing factor (Figure S1). However, the overall trends for cancer‐related mortality in the general population and diabetes‐related mortality in the general population differed significantly from cancer‐related mortality among patients with diabetes (*p* for parallelism < 0.001) (Figure S2 and Table S8).

### 3.2 Cancer‐Associated AAMR in Patients With Diabetes, Stratified by Age Group

From 1999 to 2019, the crude mortality rate per 100,000 for older adults (≥ 65 years) was vastly greater than that for middle‐aged adults (45–64 years), which was greater than that for younger adults (25–44 years). Specifically, the crude mortality rates were 0.25 (95% CI: 0.25–0.26) for the younger group, 7.64 (95% CI: 7.59–7.68) for the middle‐aged group, and 65.48 (95% CI: 65.31–65.65) for the older group (Table 1 and Table S1).

During the study period, a non‐significant increase in crude rate was seen in the younger adults group (AAPC: +0.71%; 95% CI: −0.05 to 1.53; *p* = 0.06), while a significant increase was observed in the middle‐aged adults group (AAPC: +0.95%; 95% CI: 0.79 to 1.13; *p* < 0.001). In contrast, the older adults group experienced a significant decrease in crude rates (AAPC: −0.13%; 95% CI: −0.20 to −0.04; *p* = 0.001) (Table 1 and Figure S3).

### 3.3 Cancer‐Associated AAMR in Patients With Diabetes, Stratified by Sex

Throughout the study period, the AAMR for men was consistently higher than that for women (overall AAMR for men: 20.83; 95% CI: 20.76–20.90; for women: 11.80; 95% CI: 11.75–11.84). In 1999, the AAMR for men was 20.00 (95% CI: 19.67–20.34), which increased to 20.66 (95% CI: 20.39–20.92) by 2019 (AAPC: +0.19%; 95% CI: 0.05 to 0.41; *p* = 0.02). In contrast, the AAMR for women significantly decreased from 11.84 (95% CI: 11.63–12.05) in 1999 to 11.07 (95% CI: 10.90–11.24) in 2019 (AAPC: −0.38%; 95% CI: −0.53 to −0.20; *p* < 0.001) (Table 1; Figure 1; Table S2).

### 3.4 Cancer‐Associated AAMR in Patients With Diabetes, Stratified by Race/Ethnicity

When comparing mortality rates by race/ethnicity, significant heterogeneity was seen across different groups. AAMRs were highest among NH Black or African American patients, followed by NH American Indian or Alaska Native, Hispanic or Latino, NH White and NH Asian or Pacific Islander populations (NH Black or African American: 23.72; 95% CI: 23.56–23.87; NH American Indian or Alaska Native: 21.19; 95% CI: 20.57–21.80; Hispanic or Latino: 16.39; 95% CI: 16.25–16.53; NH White: 14.60; 95% CI: 14.56–14.64; NH Asian or Pacific Islander: 11.84; 95% CI: 11.67–12.01) (Table 1 and Table S3).

**FIGURE 2:**
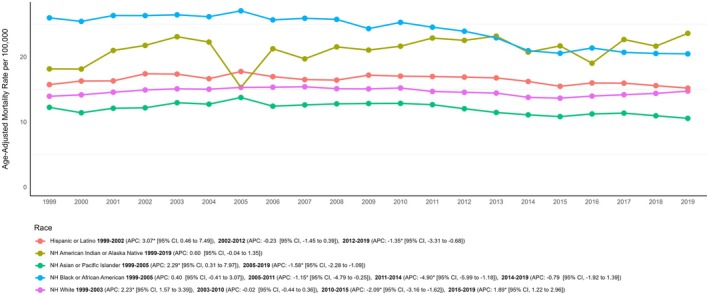
Race‐based trends in diabetes and cancer related mortality rates in adults in the United States from 1999 to 2019.

The AAMR for the NH American Indian or Alaska Native population increased the most throughout the study period (AAPC: +0.60%; 95% CI: −0.04 to 1.35; *p* = 0.06), followed by a significant increase in the NH White population (AAPC: +0.28%; 95% CI: 0.18 to 0.40; *p* < 0.001). Conversely, a significant decrease in AAMR was seen in the NH Black or African American population (AAPC: −1.17%; 95% CI: −1.37 to −0.91; *p* < 0.001), with declines also observed in the NH Asian or Pacific Islander (AAPC: −0.44%; 95% CI: −0.84 to 0.31; *p* = 0.14) and Hispanic or Latino populations (AAPC: −0.13%; 95% CI: −0.39 to 0.19; *p* = 0.39) (Table 1; Figure 2; Table S3).

### 3.5 Cancer‐Associated AAMR in Patients With Diabetes, Stratified by Geographic Region

**FIGURE 3:**
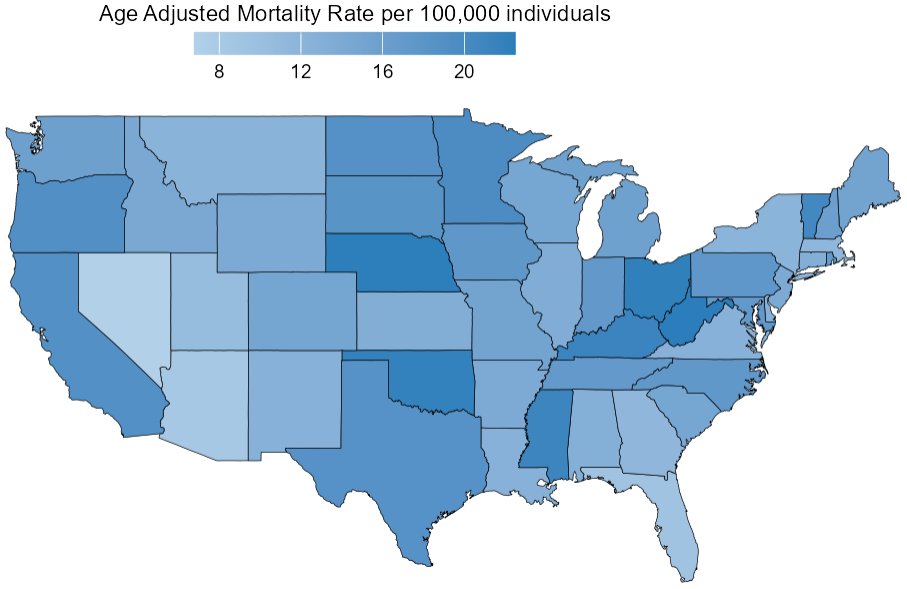
State‐wise distribution of diabetes and cancer related mortality rates in adults in the United States from 1999 to 2019.

**FIGURE 4:**
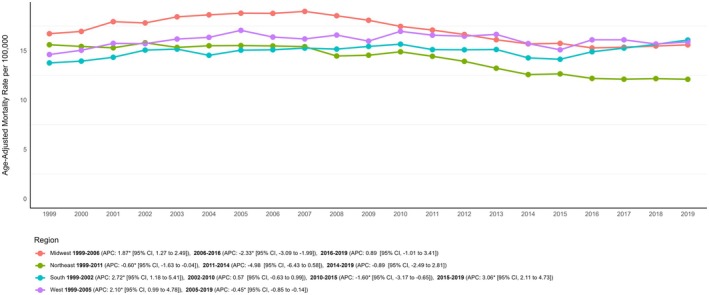
Regional trends in diabetes and cancer related mortality rates in adults in the United States from 1999 to 2019.

A prominent disparity in AAMR was observed among states, with values ranging from 6.78 (95% CI: 6.50–7.06) in Nevada to 22.49 (95% CI: 21.97–23.01) in West Virginia (Figure 4). States in the upper 90th percentile of cancer‐associated mortality in patients with diabetes included West Virginia, Nebraska, Ohio, Oklahoma, and Kentucky. These states had AAMRs that were more than twice those in the lower 10th percentile, which included Nevada, Arizona, Florida, Utah, and Massachusetts (Table S4). When comparing AAMRs across different geographic regions, the highest mortality was observed in the Midwestern region (AAMR: 17.03; 95% CI: 16.95–17.11), followed by the Western (AAMR: 16.06; 95% CI: 15.98–16.14), Southern (AAMR: 14.99; 95% CI: 14.93–15.04), and Northeastern regions (AAMR: 14.10; 95% CI: 14.02–14.17) (Table 1; Figure 3; Table S5).

Throughout the study period, non‐metropolitan areas had higher cancer‐associated mortality rates in patients with diabetes than metropolitan areas, with overall AAMRs of 18.70 (95% CI: 18.61–18.80) and 14.84 (95% CI: 14.80–14.87), respectively (Table S6). Non‐metropolitan areas experienced a significant overall increase in mortality rates (AAPC: +0.92%; 95% CI: 0.77 to 1.13; *p* < 0.001), compared to metropolitan areas, which experienced a non‐significant overall decrease (AAPC: −0.16%; 95% CI: −0.28 to 0.02; *p* = 0.07) (Table 1 and Figure S4).

### 3.6 Cancer Subtypes

When assessing mortality rates across different cancer subtypes in patients with diabetes, the highest AAMRs were observed with GI cancer (AAMR: 4.31; 95% CI: 4.29–4.33), lung cancer (AAMR: 3.08; 95% CI: 3.06–3.09) and haematological cancers (AAMR: 1.80; 95% CI: 1.79–1.82), while the lowest AAMRs were observed in prostate cancer (AAMR: 1.59, 95% CI: 1.58–1.61) and breast cancer categories (AAMR: 1.38; 95% CI: 1.37–1.39) (Table S7).

Significant changes in mortality rates were seen across all subtypes of cancers associated with diabetes during the study period. GI cancer was the only subtype to experience a significant overall increase in AAMRs (AAPC: +0.21%; 95% CI: 0.12 to 0.32; *p* < 0.001). In comparison, the other subtypes all experienced a significant decrease, with the greatest decline seen in breast cancer (AAPC: −0.84; 95% CI: −0.96 to −0.69 *p* < 0.001), followed by prostate cancer (AAPC: −0.53; 95% CI: −0.70 to −0.34 *p* < 0.001), followed by lung cancer (AAPC: −0.27%; 95% CI: 0.43 to −0.05; *p* = 0.02) (Figure S5).

## 4 Discussion

The current study provides a comprehensive analysis of mortality trends among individuals with cancer and diabetes, using CDC WONDER data from 1999 to 2019. Our findings indicate that mortality rates have significantly increased in recent years. Men consistently exhibited higher AAMRs than women, with the most pronounced effects observed in older adults (> 65). Among racial groups, Black individuals had the highest AAMR; however, the greatest rise in mortality was seen among American Indians, who surpassed Black individuals after 2014. Lastly, in terms of geographical variations, higher AAMRs were initially observed in rural areas and the Midwest, but this trend later shifted, with the West overtaking these regions (Central Illustration). Among the different types of cancer, GI cancers had the highest AAMR.

Initially, we observed a rise in cancer‐ and diabetes‐related mortality from 1999 to 2003, followed by a period of stabilisation until 2010. A steep decline in mortality trends occurred between 2010 and 2015, but this was followed by a significant increase through 2019. This pattern contrasted with diabetes‐related mortality, which remained stable during the same period. The rising trend in the early 2000s may be attributed to increasing diabetes prevalence, exacerbated by the obesity pandemic [21, 22]. The subsequent stabilisation and decline in mortality reflect the impact of several critical factors. Improvements in cancer screening and prevention have helped avert numerous cancer‐related deaths. Previous studies reported reductions in prostate cancer mortality and breast cancer mortality by 58% [23, 24]. Additionally, advancements in cancer treatment, such as improved combination chemotherapy, targeted drug therapy, and more effective radiotherapy, have contributed to declining mortality rates [25, 26]. Better glycemic control among patients with diabetes, combined with a significant increase in metformin use after 2003, has mitigated the severe consequences of diabetes [27]. Many studies have highlighted metformin's effectiveness in reducing cancer risk and improving survival among cancer patients [28, 29]. A meta‐analysis reported that metformin use was associated with longer recurrence‐free survival, overall survival, and cancer‐specific survival in patients with early‐stage cancer [29]. Furthermore, studies suggest that metformin, when combined with chemotherapy, leads to significantly higher rates of pathological complete response while reducing toxicity associated with neoadjuvant chemotherapy [29, 30]. While metformin has been associated with reduced cancer risk, newer diabetes medications, such as insulin analogs and sulfonylureas, have been linked to a potentially increased cancer risk. Curie et al. reported an increased cancer risk of 36% with sulfonylurea use and 42% with insulin use [31]. The widespread adoption of GLP‐1 receptor agonists and SGLT2 inhibitors post‐2015 may have influenced cancer trends, though their long‐term effects remain debated. While advancements in cancer treatment have extended life expectancy, they also introduce new complexities. The aging population, particularly those with long‐standing diabetes or at a higher risk of developing diabetes, faces an increased risk of cancer recurrence and worse prognoses [32].

Although our findings cannot confer causation, several studies have shown a pathophysiological association between diabetes and cancer. Elevated glucose levels enhance tumour metabolism and DNA damage, while hyperinsulinaemia and insulin‐like growth factor‐1 (IGF‐1) stimulate cancer cell proliferation. Additionally, persistent inflammation and oxidative stress contribute to tumour progression [33, 34]. A 2019 study found that hyperglycaemia damages the body's DNA by altering its structure and suppressing normal repair functions, leading to genomic instability and creating an environment conducive to cancer growth [35]. Furthermore, patients with diabetes often present with comorbid conditions such as chronic kidney disease, neuropathy, obesity, ageing, and physical inactivity, all of which may influence cancer progression [10, 36].

Our analysis identified significant gender disparities, with men exhibiting higher mortality rates from diabetes‐ and cancer‐related causes compared to women. Additionally, while mortality declined among women over the study period, men experienced a significant increase. These findings align with analyses that separately assessed diabetes and cancer prevalence in the U.S. [21, 37] However, our results contrast with previous literature suggesting that women with diabetes are at a 27% higher risk of developing cancer compared to non‐diabetic women, whereas men face a 19% increased risk [38]. Our results reveal that although women are at a greater risk of developing diabetes‐related cancer, men are more likely to suffer fatal consequences. A possible explanation for this disparity is that men are more likely to engage in unhealthy behaviours such as smoking, alcohol consumption, and drug use, which exacerbate the severity of both diabetes and cancer, thereby increasing mortality risk [39]. Additionally, genetic predisposition plays a crucial role, as sex‐steroid hormones, including higher testosterone levels in men, may contribute to increased cell growth and tumour progression [40, 41]. These findings highlight the urgent need for greater attention to men affected by diabetes and cancer. While much of the existing research focuses on the elevated cancer risk among women with diabetes, men remain a largely overlooked population despite facing significantly higher mortality rates.

We also observed significant racial disparities in AAMR. Initially, NH Black individuals had the highest AAMR; however, their mortality rate declined rapidly after 2011. In contrast, NH American Indians exhibited a significant and continuous AAMR increase over the two decades, eventually surpassing NH Black individuals. Mortality rates for NH Whites showed a slight increase, while rates for NH Asians and Hispanics remained relatively stable. This trend aligns with findings from the Strong Heart Study, which reported an increased risk of gastric, liver, and prostate cancer mortality among American Indians with diabetes [42]. The rise in deaths within this population may be attributed to the diabetes epidemic, coupled with lower rates of HbA1c control and reduced adherence to diabetic medications, both of which heighten cancer risk [43]. Furthermore, American Indians have demonstrated lower levels of basic cancer screening knowledge, a higher likelihood of presenting with advanced‐stage disease for certain screening‐detectable cancers, and more negative attitudes toward certain aspects of cancer treatment—some of which may be culturally sensitive. These factors may contribute to the disparities in cancer‐ and diabetes‐related AAMR observed in our analysis [44]. Additionally, social and environmental determinants—such as education, nutrition, exposure to environmental toxins, housing conditions, and poverty—can further exacerbate the risk of diseases like diabetes and cancer among American Indians [45]. Addressing these disparities requires a multipronged approach that includes expanding healthcare access and implementing culturally tailored interventions.

We also identified notable geographic variations in AAMR. The Midwestern region initially had the highest burden, but mortality rates steeply declined after 2006. The only region that exhibited an increase in AAMR was the South, while the Northeastern and Western regions experienced a more gradual decline. Previous studies have suggested that the Southern United States experiences a higher prevalence of diabetes [46], likely driven by socioeconomic disparities, limited access to diabetes care, and the influence of region‐specific programmes, policies, and cultural factors [47]. High obesity rates (35%–40% in states like Texas) directly increase incidence and mortality from diabetes and associated complications [48]. Southern region, also called as “cancer belt” expose residents to high levels of pollutants, increasing rates of cancer, diabetes, and cardiovascular disease [49]. These elements collectively contribute to the rising burden of both diabetes‐ and cancer‐related mortality in the region. After a period of decline, we observed a recent resurgence in AAMR across both rural and urban areas, with rural areas experiencing a disproportionately higher number of diabetes‐ and cancer‐related deaths. The increased risk of diabetes in rural regions can likely be attributed to a higher prevalence of obesity, metabolic syndrome, and greater physical inactivity [50, 51]. Additionally, rural residents are less likely to participate in diabetes self‐management education programmes, which may contribute to poorer health outcomes [52]. Limited access to specialised care, cancer screenings, and preventive healthcare programmes further exacerbate the mortality gap in these areas [53, 54, 55]. Conversely, the rise in AAMR in urban areas may be linked to factors associated with urbanisation, such as increased pollution, unhealthy lifestyles, and occupational hazards [56, 57]. To narrow the rural–urban mortality gap, increased focus on rural residents with diabetes and cancer remains essential. Expanding healthcare access, promoting preventive interventions, and enhancing disease management strategies in these communities could help mitigate the disparities.

We also observed distinct trends in individual components of cancer‐related mortality among patients with diabetes. Among the various cancer types, the highest proportion of deaths occurred due to GI cancers. Previous studies confirm a significant association between diabetes and pancreatic cancer, a type of GI malignancy [58, 59]. Mechanisms linking diabetes with GI cancer include the direct effects of hyperglycemia and a synergistic interaction between high salt intake and *Helicobacter pylori* infection. Additionally, comorbidities such as obesity, hypertension, dyslipidemia, and vascular complications may influence the development of GI cancer—either positively or negatively—through changes in lifestyle, dietary components, salt intake, and drug metabolism [60]. Lung cancer was the second most common cancer‐related cause of death among diabetes patients. It was in accordance with a meta‐analysis which found an association between diabetes and an increased risk of lung cancer [61]. However, some studies suggest that diabetes is not an independent risk factor for lung cancer, and the observed link may be confounded by smoking status [62, 63]. We also observed increased mortality in hematologic malignancies, including leukaemia, non‐Hodgkin lymphoma, and multiple myeloma, which aligns with previous literature [64, 65]. In contrast, prostate cancer‐related mortality exhibited a stable trend. The recent rise in prostate cancer‐related deaths among patients with diabetes warrants a reassessment of current research that reports a reduced prostate cancer risk in this population. Lastly, a significant reduction in breast cancer mortality was observed, likely due to increased awareness of screening programs, leading to earlier detection and improved survival rates [66].

The recent rise in diabetes and cancer‐related deaths observed in this analysis is concerning. This highlights the urgent need for more robust cancer surveillance in patients with diabetes through the implementation of opportunistic and systematic cancer screening programmes. Early identification of at‐risk individuals and timely intervention with appropriate treatments are crucial to improving outcomes. A multidisciplinary approach to diabetes management in cancer patients, incorporating a diabetes specialist, educator, and dietitian in collaboration with the cancer care team, is essential to minimise diabetes‐related complications and prevent poor treatment outcomes [67]. Cancer patients with diabetes undergoing anticancer therapy require careful management of hyperglycaemia, fluid and electrolyte balance, cardiovascular complications, and autonomic neuropathy affecting the GI tract [68]. Additionally, patient education and self‐management training, including home blood glucose monitoring, tracking treatment side effects, ensuring adherence to prescribed therapies, and ongoing education, are critical components of comprehensive diabetes care in cancer patients [69]. Furthermore, our findings suggest the need for further research into the potential benefits of metformin, with a focus on optimal dosing strategies and long‐term effects. Future longitudinal research should also explore the impact of newer anti‐diabetic medications, such as SGLT2 inhibitors and GLP‐1 agonists, on cancer risk and patient outcomes.

### 4.1 Limitations

This study has several limitations that should be acknowledged. First, the reliance on ICD‐10 codes and death certificates may have led to potential misclassification of diabetes and cancer as the underlying or contributing causes of death, potentially impacting the accuracy of mortality trends. Moreover, the CDC WONDER database does not provide detailed clinical information, such as cancer grade, stage, or comorbid conditions, which could affect diabetes‐related mortality patterns. The absence of data on specific diabetes treatments—such as types of diabetic medications and associated complications—limits the ability to assess the precise role of these therapies in increasing cancer risk and mortality. Socioeconomic determinants of health, including income, education level, and insurance status, were also not included in the dataset, which could influence access to care, treatment adherence, and outcomes. The absence of data on the timing of events also precludes mechanistic analyses, such as evaluating the role of improved imaging or cancer survival on observed trends.

## 5 Conclusion

This study highlights significant and concerning trends in diabetes‐associated cancer mortality in the U.S., with AAMR initially rising, followed by a decline, and then a renewed increase after 2015. Men were identified to be at higher risk than women. Older adults had the highest proportion of deaths; however, only middle‐aged adults experienced a significant rise in mortality. Among ethnic populations, Black individuals had the highest AAMRs while American Indians experienced a notable rise in mortality rates. Geographically, rural residents and those in the Midwestern region were disproportionately affected. Public health initiatives should prioritise improving healthcare access for high‐risk communities, increasing awareness of the link between diabetes and cancer, and promoting prevention and management strategies. Additionally, fostering healthier environments through targeted interventions is essential. Future research should focus on the long‐term impact of anti‐diabetic medications in reducing cancer risk and improving outcomes in patients with diabetes.

## Author Contributions

**Muhammad Saad:** conceptualization, Methodology, Project administration. **Dua Ali:** data curation, Investigation, Writing – review and editing. **Taimor Mohammed Khan:** software, Formal analysis, Writing – review and editing. **Ruqiat Masooma Batool:** visualization, Validation. **Muhammad Sameer Arshad:** resources, Writing – review and editing. **Peter Collins:** writing – review and editing. **Raheel Ahmed:** supervision

## Ethics Statement

The authors have nothing to report.

## Conflicts of Interest

The authors declare no conflicts of interest.

## Supporting information


**Table S1:** Diabetes and cancer‐related crude mortality rates per 100,000, stratified by age groups in the United States, 1999 to 2019.
**Table S2:** Diabetes and cancer–related age‐adjusted mortality rates per 100,000, stratified by sex in the United States, 1999 to 2019.
**Table S3:** Diabetes and Cancer–related age‐adjusted mortality rates per 100,000, stratified by race in the United States, 1999 to 2019.
**Table S4:** Diabetes and cancer–related age‐adjusted mortality rates per 100,000, stratified by states in the United States, 1999 to 2019.
**Table S5:** Diabetes and cancer–related age‐adjusted mortality rates per 100,000, stratified by census region in the United States, 1999 to 2019.
**Table S6:** Diabetes and cancer–related age‐adjusted mortality rates per 100,000, stratified by urbanisation in the United States, 1999 to 2019.
**Table S7:** Diabetes and cancer–related age‐adjusted mortality rates per 100,000, stratified by cancer subtype in the United States, 1999 to 2019.
**Table S8:** Age‐adjusted mortality rate per 100,000 for cancer vs. diabetes vs. cancer + diabetes in the United States, 1999–2019.
**Figure S1:** Cancer‐related (underlying cause) and diabetes‐related (multiple cause) age‐adjusted mortality rates per 100,000 among adults in the United States, 1999 to 2019.
**Figure S2:** Trends in age‐adjusted mortality rates for cancer, diabetes, and, cancer and diabetes among adults in the United States, 1999 to 2019.
**Figure S3:** Age‐specific trends in diabetes and cancer‐related mortality among adults in the United States, 1999 to 2019.
**Figure S4:** Trends in diabetes and cancer related mortality stratified by the level of urbanisation among adults in the United States from 1999 to 2019.
**Figure S5:** Trends in age‐adjusted mortality rates for cancer subtypes among adults with diabetes in the United States, 1999 to 2019.

## Data Availability

The data that supports the findings of this study are available in the Supporting Information—S1 of this article.
