# Supplementary material for: Trends and Disparities in Cancer‐Related Mortality Among Adults With Diabetes in the United States: 1999–2019
Source: Endocrinol Diabetes Metab. 2025 Sep 25;8(5):e70092. doi: 10.1002/edm2.70092 (PMC12463545; doi:10.1002/edm2.70092)
Supplement: Supplementary file 1 — Table S1: Diabetes and cancer‐related crude mortality rates per 100,000, stratified by age groups in the United States, 1999 to 2019. Table S2: Diabetes and cancer–related age‐adjusted mortality rates per 100,000, stratified by sex in the United States, 1999 to 2019. Table S3: Diabetes and Cancer–related age‐adjusted mortality rates per 100,000, stratified by race in the United States, 1999 to 2019. Table S4: Diabetes and cancer–related age‐adjusted mortality rates per 100,000, stratified by states in the United States, 1999 to 2019. Table S5: Diabetes and cancer–related age‐adjusted mortality rates per 100,000, stratified by census region in the United States, 1999 to 2019. Table S6: Diabetes and cancer–related age‐adjusted mortality rates per 100,000, stratified by urbanisation in the United States, 1999 to 2019. Table S7: Diabetes and cancer–related age‐adjusted mortality rates per 100,000, stratified by cancer subtype in the United States, 1999 to 2019. Table S8: Age‐adjusted mortality rate per 100,000 for cancer vs. diabetes vs. cancer + diabetes in the United States, 1999–2019. Figure S1: Cancer‐related (underlying cause) and diabetes‐related (multiple cause) age‐adjusted mortality rates per 100,000 among adults in the United States, 1999 to 2019. Figure S2: Trends in age‐adjusted mortality rates for cancer, diabetes, and, cancer and diabetes among adults in the United States, 1999 to 2019. Figure S3: Age‐specific trends in diabetes and cancer‐related mortality among adults in the United States, 1999 to 2019. Figure S4: Trends in diabetes and cancer related mortality stratified by the level of urbanisation among adults in the United States from 1999 to 2019. Figure S5: Trends in age‐adjusted mortality rates for cancer subtypes among adults with diabetes in the United States, 1999 to 2019. [file EDM2-8-e70092-s001.docx]

**Supplementary Material**

| **Crude Mortality Rate (95% CI)** | | | |
| --- | --- | --- | --- |
| **Year** | **Young Adults** | **Middle-aged Adults** | **Older Adults** |
| 1999 | 0.23 (0.20-0.26) | 7.02 (6.81-7.23) | 63.7 (62.86-64.54) |
| 2000 | 0.20 (0.17-0.23) | 6.85 (6.65-7.06) | 65.05 (64.2-65.89) |
| 2001 | 0.23 (0.20-0.26) | 6.93 (6.73-7.14) | 67.38 (66.53-68.24) |
| 2002 | 0.23 (0.20-0.26) | 7.25 (7.05-7.46) | 68.96 (68.1-69.83) |
| 2003 | 0.29 (0.25-0.32) | 7.42 (7.21-7.62) | 69.61 (68.74-70.47) |
| 2004 | 0.24 (0.20-0.27) | 7.35 (7.15-7.55) | 69.5 (68.65-70.36) |
| 2005 | 0.24 (0.20-0.27) | 7.72 (7.52-7.92) | 70.96 (70.1-71.82) |
| 2006 | 0.29 (0.26-0.33) | 7.63 (7.43-7.83) | 70.41 (69.55-71.26) |
| 2007 | 0.26 (0.22-0.29) | 7.73 (7.54-7.93) | 70.51 (69.66-71.35) |
| 2008 | 0.25 (0.22-0.29) | 7.56 (7.36-7.75) | 69.34 (68.52-70.17) |
| 2009 | 0.23 (0.20-0.27) | 7.76 (7.56-7.95) | 68.23 (67.41-69.04) |
| 2010 | 0.26 (0.22-0.29) | 8.03 (7.83-8.22) | 69.02 (68.21-69.83) |
| 2011 | 0.23 (0.20-0.27) | 7.80 (7.61-7.99) | 67.1 (66.31-67.89) |
| 2012 | 0.25 (0.21-0.28) | 7.78 (7.59-7.97) | 65.46 (64.7-66.23) |
| 2013 | 0.30 (0.26-0.33) | 7.88 (7.69-8.08) | 63.76 (63.02-64.5) |
| 2014 | 0.24 (0.21-0.27) | 7.44 (7.25-7.62) | 60.76 (60.05-61.47) |
| 2015 | 0.27 (0.23-0.30) | 7.39 (7.21-7.58) | 59.7 (59.01-60.39) |
| 2016 | 0.28 (0.24-0.31) | 7.73 (7.54-7.91) | 60.7 (60.02-61.39) |
| 2017 | 0.28 (0.24-0.31) | 7.96 (7.77-8.15) | 60.9 (60.22-61.58) |
| 2018 | 0.26 (0.23-0.30) | 8.11 (7.92-8.31) | 61.23 (60.56-61.9) |
| 2019 | 0.26 (0.22-0.29) | 8.35 (8.15-8.55) | 62.03 (61.36-62.69) |

**Supplementary Table 1.** Diabetes and cancer-related crude mortality rates per 100,000, stratified by age groups in the United States, 1999 to 2019

| **Year** | **Age-Adjusted Mortality Rate (95% CI)** | | |
| --- | --- | --- | --- |
|  | **Overall** | **Females** | **Males** |
| 1999 | 15.06 (14.88 - 15.24) | 11.84 (11.63 - 12.05) | 20 (19.67 - 20.34) |
| 2000 | 15.21 (15.03 - 15.39) | 11.96 (11.75 - 12.17) | 20.25 (19.92 - 20.59) |
| 2001 | 15.68 (15.49 - 15.86) | 12.31 (12.09 - 12.52) | 20.9 (20.56 - 21.24) |
| 2002 | 15.99 (15.81 - 16.18) | 12.57 (12.36 - 12.79) | 21.25 (20.92 - 21.59) |
| 2003 | 16.18 (16 - 16.37) | 12.58 (12.37 - 12.8) | 21.59 (21.26 - 21.93) |
| 2004 | 16.07 (15.89 - 16.25) | 12.47 (12.26 - 12.68) | 21.43 (21.1 - 21.76) |
| 2005 | 16.41 (16.23 - 16.59) | 12.76 (12.55 - 12.97) | 21.93 (21.6 - 22.26) |
| 2006 | 16.31 (16.13 - 16.49) | 12.72 (12.51 - 12.93) | 21.6 (21.27 - 21.92) |
| 2007 | 16.35 (16.18 - 16.53) | 12.62 (12.41 - 12.83) | 21.81 (21.49 - 22.13) |
| 2008 | 16.07 (15.9 - 16.25) | 12.22 (12.01 - 12.42) | 21.68 (21.36 - 21.99) |
| 2009 | 15.99 (15.82 - 16.17) | 12.21 (12.01 - 12.41) | 21.4 (21.09 - 21.71) |
| 2010 | 16.22 (16.05 - 16.39) | 12.44 (12.24 - 12.64) | 21.61 (21.3 - 21.92) |
| 2011 | 15.74 (15.58 - 15.91) | 12.11 (11.91 - 12.3) | 20.91 (20.61 - 21.21) |
| 2012 | 15.53 (15.36 - 15.69) | 11.66 (11.47 - 11.85) | 20.91 (20.62 - 21.21) |
| 2013 | 15.31 (15.15 - 15.47) | 11.53 (11.34 - 11.71) | 20.59 (20.3 - 20.88) |
| 2014 | 14.61 (14.45 - 14.77) | 10.89 (10.71 - 11.07) | 19.65 (19.37 - 19.92) |
| 2015 | 14.43 (14.27 - 14.58) | 10.78 (10.61 - 10.96) | 19.38 (19.11 - 19.66) |
| 2016 | 14.75 (14.6 - 14.91) | 10.84 (10.66 - 11.01) | 20.03 (19.76 - 20.3) |
| 2017 | 14.88 (14.73 - 15.03) | 10.8 (10.63 - 10.98) | 20.39 (20.12 - 20.66) |
| 2018 | 14.96 (14.81 - 15.11) | 10.98 (10.81 - 11.15) | 20.33 (20.06 - 20.6) |
| 2019 | 15.23 (15.08 - 15.38) | 11.07 (10.9 - 11.24) | 20.66 (20.39 - 20.92) |

**Supplementary Table 2.** Diabetes and cancer–related age-adjusted mortality rates per 100,000, stratified by sex in the United States, 1999 to 2019.

| **Year** | **Age-Adjusted Mortality Rate (95% CI)** | | | | |
| --- | --- | --- | --- | --- | --- |
|  | **NH American Indian or Alaska Native** | **NH Asian/Pacific Islander** | **NH Black/African American** | **NH White** | **Hispanic/Latino** |
| 1999 | 18.13 (14.91 - 21.35) | 12.23 (11.1 - 13.36) | 25.97 (25.14 - 26.8) | 13.93 (13.74 - 14.12) | 15.72 (14.85 - 16.59) |
| 2000 | 18.11 (15 - 21.23) | 11.4 (10.34 - 12.47) | 25.42 (24.61 - 26.24) | 14.15 (13.96 - 14.34) | 16.27 (15.41 - 17.13) |
| 2001 | 20.96 (17.65 - 24.26) | 12.09 (11.04 - 13.13) | 26.32 (25.49 - 27.14) | 14.55 (14.36 - 14.74) | 16.29 (15.46 - 17.12) |
| 2002 | 21.73 (18.37 - 25.1) | 12.16 (11.16 - 13.16) | 26.31 (25.49 - 27.13) | 14.9 (14.7 - 15.09) | 17.38 (16.54 - 18.23) |
| 2003 | 23.07 (19.67 - 26.47) | 12.94 (11.92 - 13.95) | 26.43 (25.62 - 27.24) | 15.07 (14.87 - 15.26) | 17.33 (16.51 - 18.14) |
| 2004 | 22.25 (18.96 - 25.54) | 12.72 (11.74 - 13.7) | 26.14 (25.34 - 26.94) | 15.01 (14.82 - 15.21) | 16.63 (15.85 - 17.41) |
| 2005 | 15.29 (12.65 - 17.94) | 13.74 (12.76 - 14.72) | 27.04 (26.24 - 27.85) | 15.29 (15.09 - 15.48) | 17.73 (16.95 - 18.51) |
| 2006 | 21.22 (18.14 - 24.3) | 12.41 (11.51 - 13.31) | 25.65 (24.88 - 26.43) | 15.32 (15.13 - 15.51) | 16.94 (16.2 - 17.69) |
| 2007 | 19.67 (16.75 - 22.59) | 12.6 (11.71 - 13.49) | 25.9 (25.13 - 26.67) | 15.39 (15.2 - 15.59) | 16.49 (15.78 - 17.21) |
| 2008 | 21.51 (18.52 - 24.49) | 12.77 (11.9 - 13.64) | 25.73 (24.97 - 26.49) | 15.09 (14.9 - 15.28) | 16.41 (15.71 - 17.1) |
| 2009 | 21.02 (18.14 - 23.91) | 12.82 (11.98 - 13.67) | 24.31 (23.58 - 25.04) | 15.06 (14.87 - 15.24) | 17.17 (16.48 - 17.87) |
| 2010 | 21.61 (18.7 - 24.51) | 12.84 (12.02 - 13.67) | 25.27 (24.53 - 26.01) | 15.2 (15.01 - 15.38) | 17.02 (16.34 - 17.7) |
| 2011 | 22.87 (19.92 - 25.82) | 12.64 (11.85 - 13.43) | 24.53 (23.82 - 25.24) | 14.67 (14.49 - 14.85) | 16.96 (16.31 - 17.62) |
| 2012 | 22.51 (19.68 - 25.33) | 12.02 (11.27 - 12.77) | 23.91 (23.22 - 24.6) | 14.54 (14.36 - 14.72) | 16.87 (16.23 - 17.5) |
| 2013 | 23.16 (20.36 - 25.96) | 11.44 (10.74 - 12.15) | 22.9 (22.23 - 23.56) | 14.42 (14.24 - 14.59) | 16.75 (16.13 - 17.36) |
| 2014 | 20.7 (18.19 - 23.21) | 11.08 (10.42 - 11.75) | 20.92 (20.29 - 21.54) | 13.76 (13.59 - 13.93) | 16.2 (15.62 - 16.79) |
| 2015 | 21.66 (19.09 - 24.23) | 10.81 (10.18 - 11.44) | 20.53 (19.92 - 21.14) | 13.64 (13.47 - 13.81) | 15.46 (14.9 - 16.01) |
| 2016 | 19 (16.69 - 21.32) | 11.22 (10.59 - 11.85) | 21.34 (20.73 - 21.95) | 13.96 (13.79 - 14.13) | 15.97 (15.41 - 16.52) |
| 2017 | 22.64 (20.18 - 25.09) | 11.34 (10.72 - 11.95) | 20.67 (20.08 - 21.25) | 14.17 (14 - 14.34) | 15.94 (15.4 - 16.48) |
| 2018 | 21.62 (19.29 - 23.95) | 10.94 (10.35 - 11.53) | 20.5 (19.92 - 21.07) | 14.37 (14.2 - 14.54) | 15.56 (15.04 - 16.08) |
| 2019 | 23.59 (21.19 - 25.99) | 10.54 (9.98 - 11.1) | 20.44 (19.87 - 21.01) | 14.72 (14.55 - 14.89) | 15.18 (14.68 - 15.69) |

**Supplementary Table 3.** Diabetes and Cancer–related age-adjusted mortality rates per 100,000, stratified by race in the United States, 1999 to 2019.

| **State** | **Age-Adjusted Mortality Rate [95% CI]** |
| --- | --- |
| Alabama | 12.96 (12.69 - 13.22) |
| Alaska | 13.58 (12.59 - 14.57) |
| Arizona | 8.32 (8.13 - 8.5) |
| Arkansas | 14.02 (13.67 - 14.37) |
| California | 18.92 (18.8 - 19.05) |
| Colorado | 14.92 (14.61 - 15.22) |
| Connecticut | 13.18 (12.88 - 13.48) |
| Delaware | 12.91 (12.31 - 13.51) |
| District of Columbia | 17.8 (16.87 - 18.73) |
| Florida | 9.32 (9.22 - 9.42) |
| Georgia | 11.45 (11.26 - 11.65) |
| Hawaii | 14.66 (14.15 - 15.17) |
| Idaho | 14.3 (13.78 - 14.81) |
| Illinois | 13.55 (13.38 - 13.72) |
| Indiana | 17.29 (17.02 - 17.56) |
| Iowa | 17.55 (17.18 - 17.91) |
| Kansas | 13.24 (12.89 - 13.58) |
| Kentucky | 21.22 (20.86 - 21.58) |
| Louisiana | 12.63 (12.35 - 12.9) |
| Maine | 15.16 (14.66 - 15.66) |
| Maryland | 17.2 (16.91 - 17.49) |
| Massachusetts | 11.61 (11.4 - 11.81) |
| Michigan | 15.7 (12.69 - 13.22) |
| Minnesota | 19.68 (12.59 - 14.57) |
| Mississippi | 21.18 (8.13 - 8.5) |
| Missouri | 15.16 (13.67 - 14.37) |
| Montana | 12.34 (18.8 - 19.05) |
| Nebraska | 22.38 (14.61 - 15.22) |
| Nevada | 6.78 (12.88 - 13.48) |
| New Hampshire | 16.03 (12.31 - 13.51) |
| New Jersey | 13.89 (16.87 - 18.73) |
| New Mexico | 12.56 (9.22 - 9.42) |
| New York | 12.05 (11.26 - 11.65) |
| North Carolina | 17.44 (14.15 - 15.17) |
| North Dakota | 18.69 (13.78 - 14.81) |
| Ohio | 22.11 (13.38 - 13.72) |
| Oklahoma | 21.9 (17.02 - 17.56) |
| Oregon | 19.1 (17.18 - 17.91) |
| Pennsylvania | 17.54 (12.89 - 13.58) |
| Rhode Island | 17.09 (20.86 - 21.58) |
| South Carolina | 14.78 (12.35 - 12.9) |
| South Dakota | 18.23 (14.66 - 15.66) |
| Tennessee | 17.18 (16.91 - 17.49) |
| Texas | 18.56 (11.4 - 11.81) |
| Utah | 10.68 (10.3 - 11.07) |
| Vermont | 20.41 (19.53 - 21.3) |
| Virginia | 12.63 (12.41 - 12.84) |
| Washington | 15.87 (15.61 - 16.13) |
| West Virginia | 22.49 (21.97 - 23.01) |
| Wisconsin | 14.8 (14.54 - 15.05) |
| Wyoming | 13.99 (13.15 - 14.82) |

**Supplementary Table 4.** Diabetes and cancer–related age-adjusted mortality rates per 100,000, stratified by states in the United States, 1999 to 2019.

| **Year** | **Age-Adjusted Mortality Rate (95% CI)** | | | |
| --- | --- | --- | --- | --- |
|  | **Northeast** | **Midwest** | **South** | **West** |
| 1999 | 15.6 (15.2 - 16.01) | 16.72 (16.33 - 17.11) | 13.76 (13.47 - 14.05) | 14.61 (14.21 - 15.01) |
| 2000 | 15.43 (15.03 - 15.83) | 16.95 (16.55 - 17.34) | 13.94 (13.65 - 14.23) | 15.04 (14.63 - 15.44) |
| 2001 | 15.29 (14.9 - 15.68) | 17.94 (17.53 - 18.34) | 14.33 (14.04 - 14.62) | 15.74 (15.33 - 16.14) |
| 2002 | 15.8 (15.4 - 16.2) | 17.81 (17.42 - 18.21) | 15.06 (14.76 - 15.36) | 15.71 (15.31 - 16.11) |
| 2003 | 15.33 (14.94 - 15.72) | 18.43 (18.02 - 18.83) | 15.16 (14.87 - 15.46) | 16.18 (15.78 - 16.58) |
| 2004 | 15.5 (15.11 - 15.89) | 18.63 (18.23 - 19.04) | 14.53 (14.24 - 14.82) | 16.35 (15.95 - 16.75) |
| 2005 | 15.52 (15.13 - 15.91) | 18.8 (18.4 - 19.2) | 15.05 (14.76 - 15.34) | 17.06 (16.65 - 17.46) |
| 2006 | 15.48 (15.09 - 15.86) | 18.78 (18.38 - 19.18) | 15.09 (14.8 - 15.38) | 16.38 (15.99 - 16.78) |
| 2007 | 15.41 (15.02 - 15.79) | 18.98 (18.58 - 19.38) | 15.26 (14.97 - 15.54) | 16.19 (15.8 - 16.58) |
| 2008 | 14.46 (14.09 - 14.83) | 18.54 (18.14 - 18.93) | 15.16 (14.88 - 15.44) | 16.58 (16.19 - 16.97) |
| 2009 | 14.54 (14.17 - 14.91) | 18.08 (17.69 - 18.46) | 15.43 (15.15 - 15.71) | 15.97 (15.6 - 16.35) |
| 2010 | 14.89 (14.52 - 15.26) | 17.46 (17.08 - 17.84) | 15.66 (15.38 - 15.94) | 16.96 (16.58 - 17.34) |
| 2011 | 14.43 (14.07 - 14.8) | 17.09 (16.72 - 17.46) | 15.11 (14.84 - 15.38) | 16.57 (16.19 - 16.94) |
| 2012 | 13.92 (13.57 - 14.28) | 16.64 (16.28 - 17) | 15.09 (14.82 - 15.35) | 16.47 (16.11 - 16.84) |
| 2013 | 13.22 (12.88 - 13.56) | 16.11 (15.75 - 16.46) | 15.12 (14.85 - 15.38) | 16.65 (16.29 - 17.02) |
| 2014 | 12.58 (12.25 - 12.92) | 15.69 (15.34 - 16.03) | 14.27 (14.02 - 14.53) | 15.72 (15.38 - 16.07) |
| 2015 | 12.65 (12.32 - 12.98) | 15.74 (15.39 - 16.08) | 14.13 (13.89 - 14.38) | 15.09 (14.76 - 15.43) |
| 2016 | 12.19 (11.87 - 12.52) | 15.28 (14.94 - 15.61) | 14.89 (14.64 - 15.14) | 16.1 (15.76 - 16.44) |
| 2017 | 12.11 (11.79 - 12.42) | 15.35 (15.02 - 15.68) | 15.26 (15.01 - 15.51) | 16.1 (15.77 - 16.44) |
| 2018 | 12.17 (11.85 - 12.48) | 15.47 (15.14 - 15.8) | 15.64 (15.39 - 15.88) | 15.68 (15.35 - 16) |
| 2019 | 12.1 (11.79 - 12.42) | 15.59 (15.26 - 15.91) | 16.08 (15.83 - 16.33) | 15.88 (15.55 - 16.2) |

**Supplementary Table 5.** Diabetes and cancer–related age-adjusted mortality rates per 100,000, stratified by census region in the United States, 1999 to 2019.

| **Year** | **Age-Adjusted Mortality Rate (95% CI)** | |
| --- | --- | --- |
|  | **Metropolitan** | **Non-metropolitan** |
| 1999 | 14.66 (14.46 - 14.86) | 16.6 (16.17 - 17.04) |
| 2000 | 14.77 (14.57 - 14.97) | 17.07 (16.62 - 17.51) |
| 2001 | 15.05 (14.85 - 15.24) | 18.32 (17.86 - 18.77) |
| 2002 | 15.44 (15.24 - 15.64) | 18.52 (18.06 - 18.98) |
| 2003 | 15.53 (15.33 - 15.72) | 19.04 (18.59 - 19.5) |
| 2004 | 15.43 (15.23 - 15.63) | 19.03 (18.57 - 19.49) |
| 2005 | 15.76 (15.57 - 15.96) | 19.45 (18.99 - 19.91) |
| 2006 | 15.66 (15.47 - 15.86) | 19.23 (18.78 - 19.69) |
| 2007 | 15.66 (15.47 - 15.85) | 19.44 (18.99 - 19.89) |
| 2008 | 15.39 (15.2 - 15.57) | 19.34 (18.89 - 19.79) |
| 2009 | 15.29 (15.1 - 15.47) | 19.16 (18.72 - 19.61) |
| 2010 | 15.52 (15.33 - 15.7) | 19.45 (19 - 19.89) |
| 2011 | 15.13 (14.95 - 15.31) | 18.6 (18.17 - 19.03) |
| 2012 | 14.9 (14.72 - 15.07) | 18.55 (18.12 - 18.97) |
| 2013 | 14.7 (14.52 - 14.87) | 18.26 (17.84 - 18.68) |
| 2014 | 13.97 (13.8 - 14.13) | 17.66 (17.25 - 18.07) |
| 2015 | 13.68 (13.51 - 13.84) | 18.05 (17.64 - 18.46) |
| 2016 | 14.06 (13.9 - 14.23) | 18.3 (17.89 - 18.72) |
| 2017 | 14.04 (13.87 - 14.2) | 19.32 (18.89 - 19.74) |
| 2018 | 14.04 (13.88 - 14.2) | 19.64 (19.22 - 20.06) |
| 2019 | 14.27 (14.12 - 14.43) | 19.85 (19.43 - 20.26) |

**Supplementary Table 6.** Diabetes and cancer–related age-adjusted mortality rates per 100,000, stratified by urbanization in the United States, 1999 to 2019.

| **Year** | **Age-Adjusted Mortality Rate (95% CI)** | | | | |
| --- | --- | --- | --- | --- | --- |
|  | **Lung** | **Gastrointestinal** | **Prostate** | **Breast** | **Hematologic Malignancies** |
| 1999 | 2.87 (2.79 - 2.95) | 4.17 (4.07 - 4.26) | 1.69 (1.63 - 1.75) | 1.46 (1.4 - 1.52) | 1.75 (1.69 - 1.81) |
| 2000 | 3 (2.92 - 3.09) | 4.21 (4.12 - 4.31) | 1.7 (1.64 - 1.76) | 1.47 (1.42 - 1.53) | 1.69 (1.63 - 1.75) |
| 2001 | 3.18 (3.1 - 3.27) | 4.27 (4.17 - 4.36) | 1.75 (1.69 - 1.81) | 1.49 (1.43 - 1.55) | 1.78 (1.72 - 1.84) |
| 2002 | 3.22 (3.13 - 3.3) | 4.41 (4.32 - 4.51) | 1.74 (1.68 - 1.8) | 1.51 (1.45 - 1.57) | 1.78 (1.72 - 1.84) |
| 2003 | 3.33 (3.25 - 3.41) | 4.41 (4.32 - 4.51) | 1.7 (1.64 - 1.76) | 1.5 (1.44 - 1.55) | 1.85 (1.79 - 1.92) |
| 2004 | 3.26 (3.18 - 3.34) | 4.43 (4.33 - 4.52) | 1.71 (1.65 - 1.77) | 1.5 (1.45 - 1.56) | 1.85 (1.79 - 1.91) |
| 2005 | 3.39 (3.31 - 3.47) | 4.5 (4.4 - 4.59) | 1.73 (1.68 - 1.79) | 1.49 (1.44 - 1.55) | 1.88 (1.82 - 1.94) |
| 2006 | 3.36 (3.28 - 3.44) | 4.5 (4.41 - 4.6) | 1.63 (1.58 - 1.69) | 1.5 (1.45 - 1.56) | 1.87 (1.81 - 1.93) |
| 2007 | 3.43 (3.34 - 3.51) | 4.46 (4.36 - 4.55) | 1.69 (1.63 - 1.75) | 1.45 (1.4 - 1.51) | 1.85 (1.79 - 1.91) |
| 2008 | 3.33 (3.25 - 3.41) | 4.44 (4.35 - 4.53) | 1.7 (1.64 - 1.75) | 1.41 (1.36 - 1.46) | 1.82 (1.77 - 1.88) |
| 2009 | 3.38 (3.3 - 3.46) | 4.34 (4.25 - 4.43) | 1.64 (1.59 - 1.7) | 1.41 (1.36 - 1.47) | 1.84 (1.78 - 1.9) |
| 2010 | 3.35 (3.28 - 3.43) | 4.42 (4.33 - 4.51) | 1.62 (1.56 - 1.67) | 1.43 (1.37 - 1.48) | 1.89 (1.83 - 1.95) |
| 2011 | 3.2 (3.12 - 3.28) | 4.31 (4.22 - 4.4) | 1.56 (1.51 - 1.61) | 1.37 (1.32 - 1.42) | 1.81 (1.75 - 1.87) |
| 2012 | 3.14 (3.07 - 3.22) | 4.23 (4.14 - 4.31) | 1.58 (1.52 - 1.63) | 1.33 (1.28 - 1.38) | 1.87 (1.81 - 1.92) |
| 2013 | 3.08 (3.01 - 3.16) | 4.24 (4.15 - 4.32) | 1.54 (1.49 - 1.59) | 1.31 (1.27 - 1.36) | 1.77 (1.71 - 1.82) |
| 2014 | 2.83 (2.76 - 2.9) | 4.13 (4.04 - 4.21) | 1.42 (1.37 - 1.47) | 1.24 (1.19 - 1.28) | 1.72 (1.66 - 1.77) |
| 2015 | 2.76 (2.69 - 2.83) | 4.07 (3.99 - 4.16) | 1.41 (1.36 - 1.45) | 1.2 (1.16 - 1.24) | 1.73 (1.68 - 1.78) |
| 2016 | 2.77 (2.7 - 2.83) | 4.21 (4.13 - 4.29) | 1.44 (1.39 - 1.49) | 1.23 (1.19 - 1.28) | 1.74 (1.69 - 1.79) |
| 2017 | 2.77 (2.71 - 2.84) | 4.23 (4.15 - 4.31) | 1.49 (1.44 - 1.54) | 1.22 (1.17 - 1.26) | 1.72 (1.67 - 1.77) |
| 2018 | 2.73 (2.67 - 2.8) | 4.29 (4.21 - 4.37) | 1.5 (1.46 - 1.55) | 1.21 (1.17 - 1.26) | 1.81 (1.76 - 1.86) |
| 2019 | 2.76 (2.69 - 2.82) | 4.38 (4.3 - 4.46) | 1.55 (1.5 - 1.6) | 1.25 (1.21 - 1.29) | 1.8 (1.75 - 1.86) |

**Supplementary Table 7.** Diabetes and cancer–related age-adjusted mortality rates per 100,000, stratified by cancer subtype in the United States, 1999 to 2019.

| **Age-Adjusted Mortality Rate (95% CI)** | | | |
| --- | --- | --- | --- |
| **Year** | **Cancer** | **Diabetes** | **Cancer + Diabetes** |
| 1999 | 343.73 (342.87-344.6) | 118.47 (117.96-118.97) | 15.06 (14.88-15.24) |
| 2000 | 340.83 (339.98-341.69) | 118.99 (118.48-119.49) | 15.21 (15.03-15.39) |
| 2001 | 335.04 (334.2-335.88) | 119.81 (119.31-120.31) | 15.68 (15.49-15.86) |
| 2002 | 331.06 (330.23-331.89) | 121.12 (120.61-121.62) | 15.99 (15.81-16.18) |
| 2003 | 324.64 (323.83-325.46) | 120.94 (120.44-121.44) | 16.18 (16.00-16.37) |
| 2004 | 317.07 (316.27-317.87) | 117.99 (117.5-118.48) | 16.07 (15.89-16.25) |
| 2005 | 314.15 (313.36-314.94) | 119.9 (119.41-120.39) | 16.41 (16.23-16.59) |
| 2006 | 308.23 (307.46-309) | 116.32 (115.84-116.79) | 16.31 (16.13-16.49) |
| 2007 | 303.26 (302.5-304.02) | 114.24 (113.77-114.71) | 16.35 (16.18-16.53) |
| 2008 | 298.54 (297.79-299.29) | 113.04 (112.58-113.5) | 16.07 (15.90-16.25) |
| 2009 | 292.88 (292.15-293.62) | 109.33 (108.88-109.78) | 15.99 (15.82-16.17) |
| 2010 | 291.71 (290.99-292.44) | 109.13 (108.68-109.57) | 16.22 (16.05-16.39) |
| 2011 | 284.66 (283.95-285.37) | 108.58 (108.14-109.02) | 15.74 (15.58-15.91) |
| 2012 | 280.74 (280.04-281.44) | 106.74 (106.31-107.17) | 15.53 (15.36-15.69) |
| 2013 | 275.17 (274.49-275.85) | 106.75 (106.32-107.17) | 15.31 (15.15-15.47) |
| 2014 | 271.34 (270.67-272.01) | 103.52 (103.1-103.93) | 14.61 (14.45-14.77) |
| 2015 | 267.08 (266.42-267.74) | 104.39 (103.98-104.81) | 14.43 (14.27-14.58) |
| 2016 | 262.96 (262.31-263.6) | 104.65 (104.24-105.05) | 14.75 (14.60-14.91) |
| 2017 | 258.35 (257.71-258.98) | 106.88 (106.47-107.29) | 14.88 (14.73-15.03) |
| 2018 | 253.54 (252.92-254.16) | 106.6 (106.2-107.01) | 14.96 (14.81-15.11) |
| 2019 | 249.2 (248.59-249.81) | 107 (106.6-107.4) | 15.23 (15.08-15.38) |

**Supplementary Table 8.** Age-adjusted mortality rate per 100,000 for cancer vs. diabetes vs. cancer + diabetes in the United States, 1999-2019


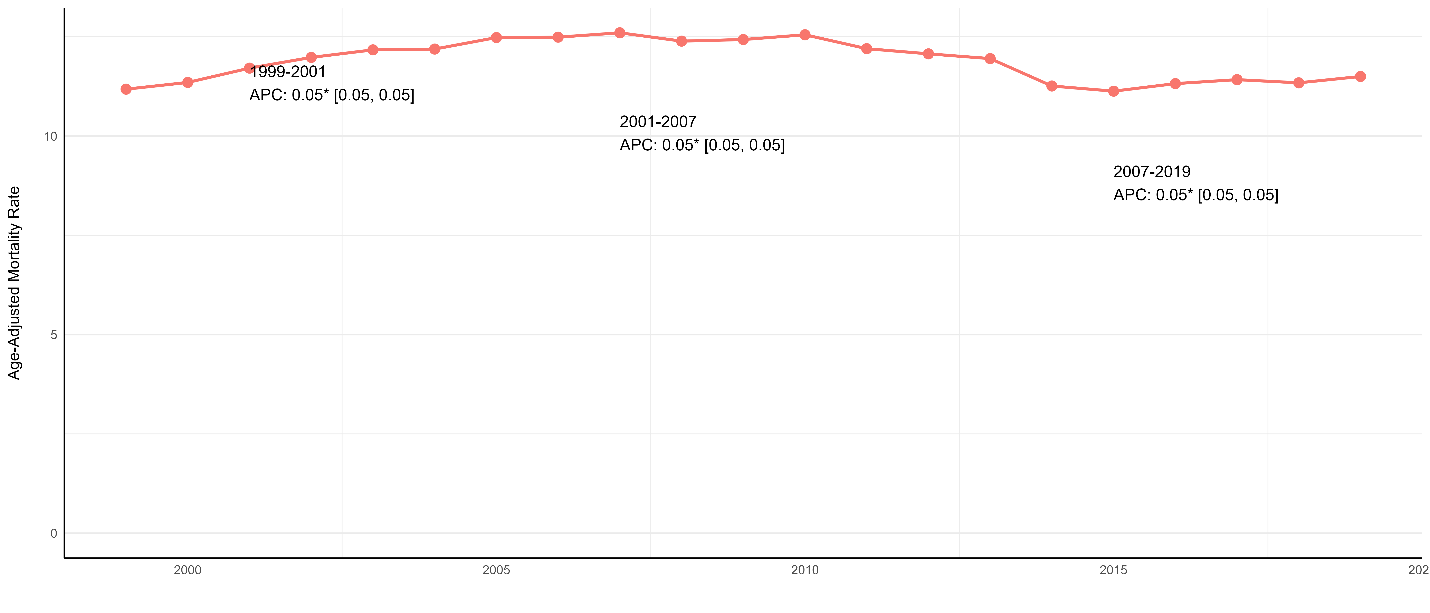


**Supplementary Figure 1. Cancer-related (underlying cause) and diabetes-related (multiple cause) age-adjusted mortality rates per 100,000 among adults in the United States, 1999 to 2019.**


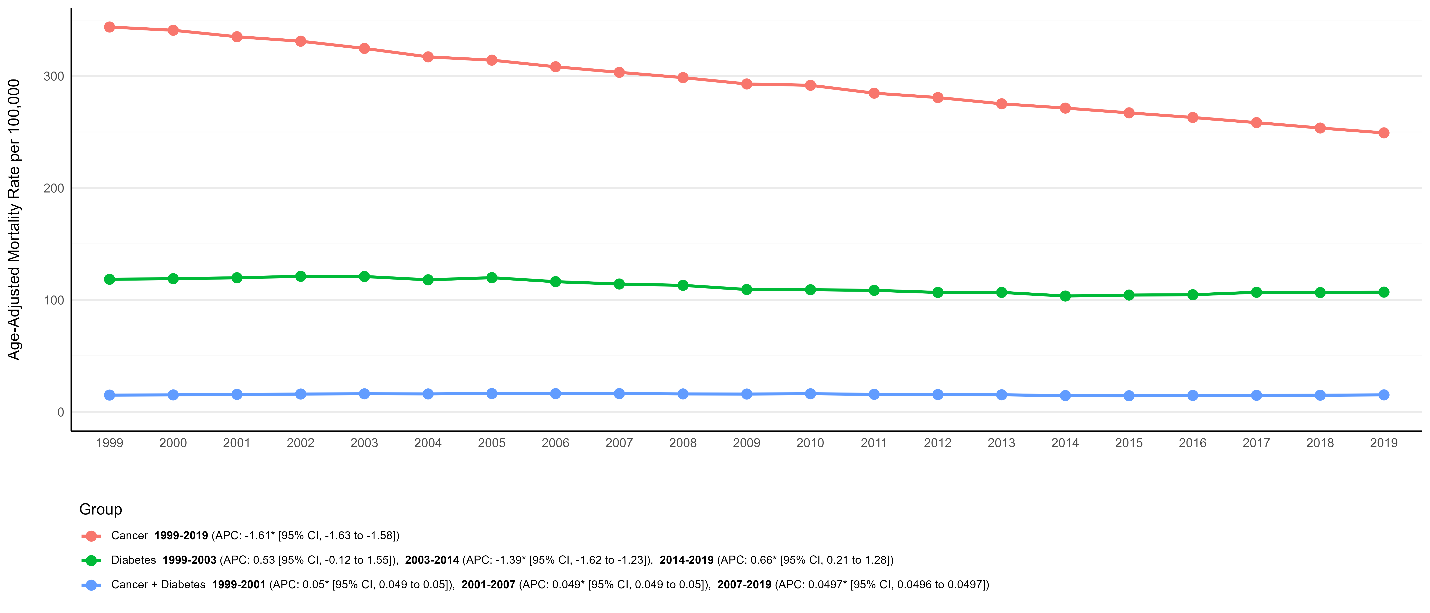


**Supplementary Figure 2. Trends in age-adjusted mortality rates for cancer, diabetes, and, cancer and diabetes among adults in the United States, 1999 to 2019.**


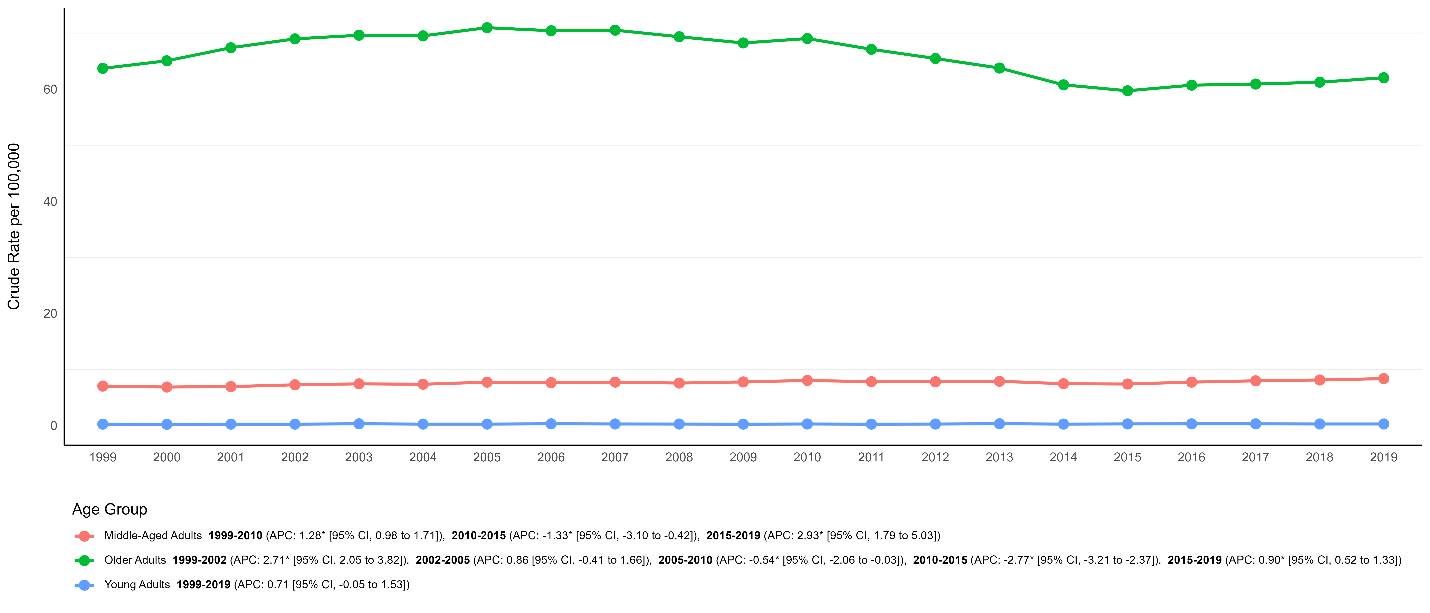


**Supplementary Figure 3.** Age-specific trends in diabetes and cancer-related mortality among adults in the United States, 1999 to 2019.


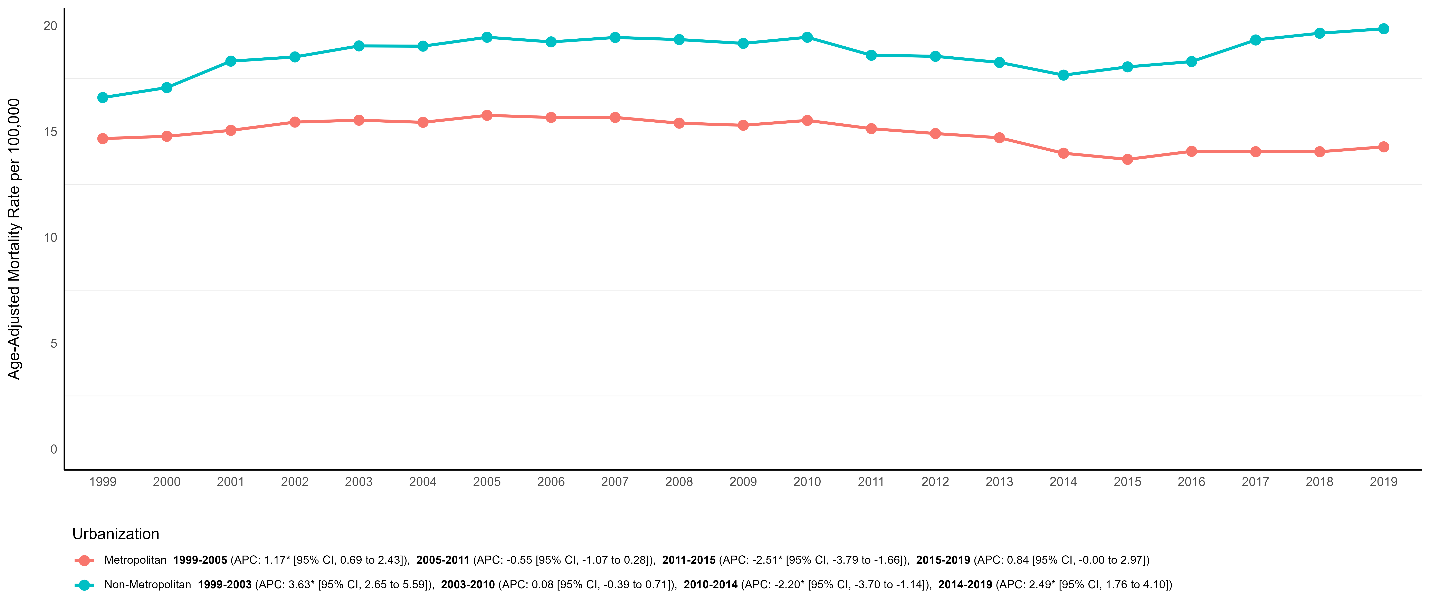


**Supplementary Figure 4.** Trends in diabetes and cancer related mortality stratified by the level of urbanization among adults in the United States from 1999 to 2019.


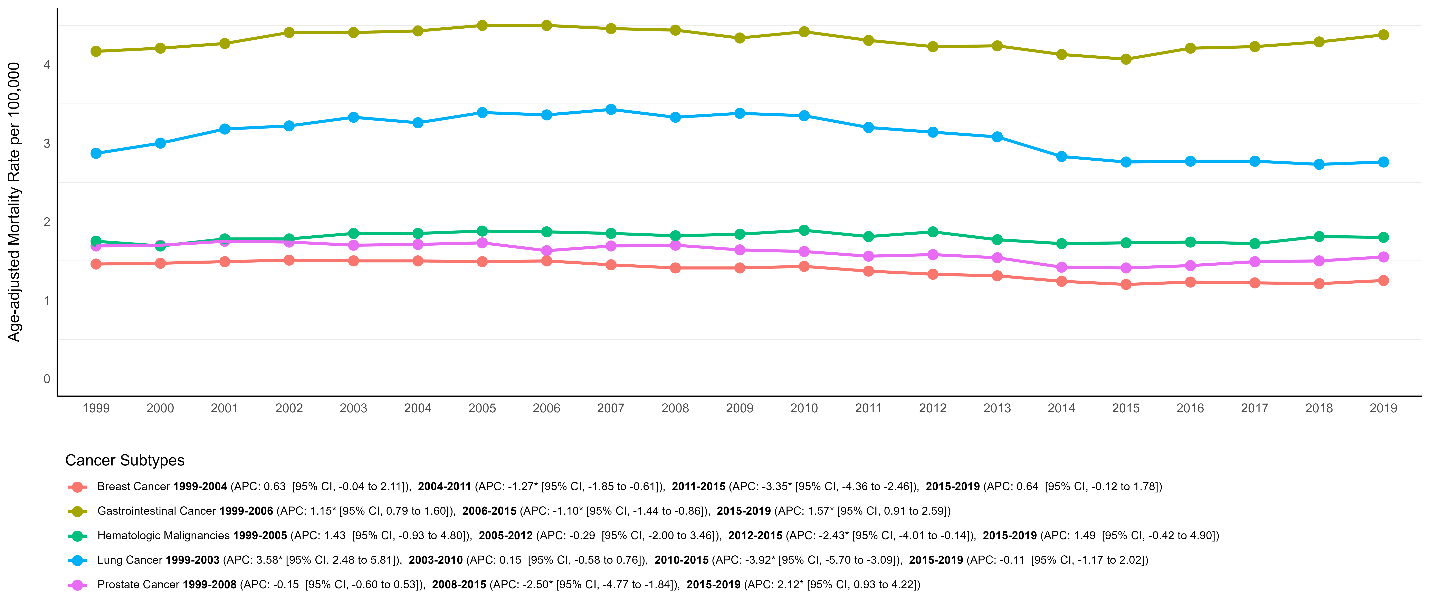


**Supplementary Figure 5.** Trends in age-adjusted mortality rates for cancer subtypes among adults with diabetes in the United States, 1999 to 2019.
